# Larger Head Displacement to Optic Flow Presented in the Lower Visual Field

**DOI:** 10.1177/2041669519886903

**Published:** 2019-11-22

**Authors:** Kanon Fujimoto, Hiroshi Ashida

**Affiliations:** Department of Psychology, Graduate School of Letters, Kyoto University, Japan

**Keywords:** postural control, visual field, optic flow, self-motion, vection

## Abstract

Optic flow that simulates self-motion often produces postural adjustment. Although literature has suggested that human postural control depends largely on visual inputs from the lower field in the environment, effects of the vertical location of optic flow on postural responses are not well investigated. Here, we examined whether optic flow presented in the lower visual field produces stronger responses than optic flow in the upper visual field. Either expanding or contracting optic flow was presented in upper, lower, or full visual fields through an Oculus Rift head-mounted display. Head displacement and vection strength were measured. Results showed larger head displacement under the optic flow presentation in the full visual field and the lower visual field than the upper visual field, during early period of presentation of the contracting optic flow. Vection was strongest in the full visual field and weakest in the upper visual field. Our findings of lower field superiority in head displacement and vection support the notion that ecologically relevant information has a particularly important role in human postural control and self-motion perception.

## Introduction

Postural control in humans requires multisensory information including visual, vestibular, and proprioceptive inputs (Bronstein, 1986; Day, Séverac Cauquil, Bartolomei, Pastor, & Lyon, 1997; Dichgans, Held, Young, & Brandt, 1972; Hwang, Agada, Kiemel, & Jeka, 2014; Lee & Lishman, 1977; Lestienne, Soechting, & Berthoz, 1977; van Asten, Gielen, & Denier van der Gon, 1988a, 1988b). Vision in particular plays an important role in controlling posture. Postural sway largely increases when visual information is impoverished (A. S. Edwards, 1946; Paulus, Straube, & Brandt, 1984). Standing in a challenging posture without vision is difficult (Lee & Lishman, 1977). Moreover, when visual stimulation simulating self-motion, known as optic flow, is presented in a large area of the visual field, a standing observer often sways in the same direction as visual motion (visually evoked postural response [VEPR]; Berthoz, Lacour, Soechting, & Vidal, 1979; Dichgans & Brandt, 1978; Lee & Aronson, 1974; Lee & Lishman, 1975; Lestienne et al., 1977; Stoffregen, 1985; van Asten et al., 1988a, 1988b) to stabilize balance of the body (Lestienne et al., 1977; Lishman & Lee, 1973).

Biases in visual processing across the upper and lower visual fields have been reported, suggesting an ecological influence on visual processing. While some studies associate lower visual-field superiority with visuomotor control in peripersonal space (Danckert & Goodale, 2001; Previc, 1990; Zito, Cazzoli, Müri, Mosimann, & Nef, 2016), others attribute it to a more locomotion-specific perspective (Dobkins & Rezec, 2004; Gibson, 1950; Previc, 1998; Skrandies, 1987), as objects near the ground are more stable and therefore provide more reliable information on self-motion than those nearer the sky (Gibson, 1950). More specifically, superiority of the lower versus upper visual field has been shown in the visuomotor control of upper (Danckert & Goodale, 2001) and lower (Graci, 2011; Timmis, Bennett, & Buckley, 2009) limbs, in brain electrical activities to visual stimulation (Amenedo, Pazo-Alvarez, & Cadaveira, 2007; Kremláček, Kuba, Chlubnová, & Kubová, 2004; Skrandies, 1987), ganglion cell densities (Croner & Kaplan, 1995; Curcio & Allen, 1990), attentional bias (Dobkins & Rezec, 2004; He, Cavanagh, & Intriligator, 1996), shape perception (Schmidtmann, Logan, Kennedy, Gordon, & Loffler, 2015), spatiotemporal perception (Carrasco, Wei, Yeshurun, & Orduña, 1998; Carrasco, Williams, & Yeshurun, 2006; Previc, 1990; Talgar & Carrasco, 2002), and motion perception (Bilodeau & Faubert, 1997; M. Edwards & Badcock, 1993; Lakha & Humphreys, 2005; Raymond, 1994; Zito et al., 2016), although one study showed superior depth sensitivity in the upper visual field (Levine & McAnany, 2005).

Concerning self-motion perception (i.e., vection; Fischer & Kornmüller, 1930), research is clear regarding the dominant role of visual inputs from the lower field (D’Avossa & Kersten, 1996; Riecke, 2010; Sato, Seno, Kanaya, & Hukazawa, 2007; Telford & Frost, 1993; Trutoiu, Mohler, Schulte-Pelkum, & Bülthoff, 2009). Tamada and Seno (2015) showed substantial illusion of self-motion, as well as postural sway, from the floor projection of optic flow. They also reported an influence of stimulus size and speed on vection and postural sway, consistent with the previous studies using frontal projection (Berthoz, Pavard, & Young, 1975; Brandt, Dichgans, & Koenig, 1973; Lestienne et al., 1977). Optic flow in the lower visual field was reported to induce a stronger vection than optic flow in the upper visual field (Sato et al., 2007; Telford & Frost, 1993). Heading direction estimation is also more precise when optic flow is presented in lower visual field compared with the upper visual field (D’Avossa & Kersten, 1996).

Although several lines of research point toward dominance of visual inputs from the lower field for postural control (Gibson, 1950; Previc, 1998), the phenomenon remains to be confirmed experimentally. Some studies presented optic flow stimuli only on the ground, assuming that this is the most effective visual stimulation for postural sway, but they did not directly examine the influence of optic flow location (Agathos, Bernardin, Baranton, Assaiante, & Isableu, 2017; Baumberger, Isableu, & Flückiger, 2004; Flückiger & Baumberger, 1988; Tamada & Seno, 2015). Flückiger and Baumberger (1988) examined postural response to optic flow at ground level and compared their results with a study that examined postural response to the visual motion of an optical tunnel (Lestienne et al., 1977). They reported that ground-level optic flow produced relatively rapid postural response compared with that found by Lestienne et al. (1977). However, various factors including texture patterns, flow velocities, acceleration rates, stimulus sizes, and experimental situations differed between these studies, leaving unclear the specific effect of visual location of the optic flow on postural response.

In this study, we investigated whether optic flow presented in the lower visual field indeed has a predominant role in postural control. To this end, we measured head movement under the presentation of optic flow in either the upper or lower visual field. Although postural sway studies often measure center of foot pressure (CoP) sway and body segment movements, we measured only head movement, partly because it was readily obtained by an Oculus Rift virtual reality (VR) head-mounted display (HMD; Oculus VR, Irvine, CA, USA) and because of our limited equipment, but also because we wanted to minimize participants’ awareness of the body sway recording and to measure their postural responses more naturally. We also note that some studies have measured head and CoP sway concurrently, finding that they moved in the same manner to presentation of uniform visual roll motion (Tanahashi, Ujike, Kozawa, & Ukai, 2007) and lateral translation (Guerraz & Bronstein, 2008). Our preliminary results for concurrent measurement also supported this for radial motion. Importantly, the upper parts of the body modulate the magnitude of VEPR (Kawakita, Kuno, Miyake, & Watanabe, 2000; Kuno, Kawakita, Kawakami, Miyake, & Watanabe, 1999) and therefore appear suitable for measuring the effect of visual stimuli on postural sway. We hypothesized that optic flow presented in the lower visual field would yield larger VEPR than upper visual field presentation because objects near the ground are more ecologically reliable for perceiving self-motion and controlling posture. We also measured full-field and hemifield (upper or lower) optic flows. In addition to head movement, we measured perceived vection to check consistency of postural sway with the perception of self-motion (Kawakita et al., 2000; Kuno et al., 1999; Lestienne et al., 1977; Lubeck, Bos, & Stins, 2015), given that this relationship has been challenged (Previc, 1992; Previc & Mullen, 1991).

## Methods

### Participants

A total of nine adult volunteers (four females and five males; mean age = 22.11 years; *SD* = 1.69) took part in the experiment, all recruited from Kyoto University. All participants had normal or corrected-to-normal visual acuity with contact lenses, excluding the possibility of glasses distorting participants’ vertical field of view (FoV). None had a history of vestibular disorders. They were informed about the purpose of the study and gave written consent for the procedure, which was in accordance with the ethics standards of the Declaration of Helsinki and approved by the local ethics committee of Kyoto University.

### Apparatus

Participants observed visual stimuli on an Oculus Rift CV1 HMD, which has a pair of organic LED displays with a resolution of 1,080 × 1,200 pixels/eye and a refresh rate of 90 Hz. The FoV of the display was approximately 110° visual angle diagonally. Lens distance of the HMD was corrected to each participant’s interpupillary distance. HMD position and orientation were recorded at the rate of 50 Hz by using the tracking system of the Rift. The experiment was controlled by a PC running Windows 10 (Microsoft, Redmond, WA, USA). An Xbox One gamepad (Microsoft) was used to collect participants’ responses.

### Stimuli

**Figure 1.**
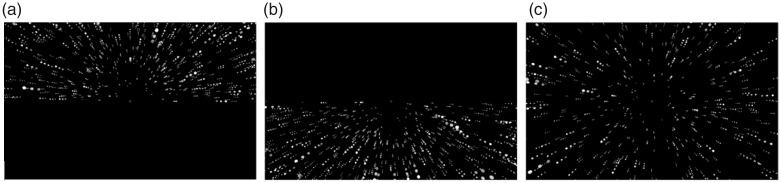
Optic flows generated on the displays by the moving viewpoint. Spheres are distributed within (a) upper, (b) lower, or (c) full area of the VR scene. Note that the actual visual stimuli were viewed with binocular disparities.

VR scenes were created with the 3D Unity engine 5.3.1 (Unity Technologies, San Francisco, CA, USA). A virtual cloud composed of 1,500 white spheres (each 0.1 m in diameter in the scene) was presented against a black background in the test scene as follows. Cloud structure differed by flow location. In the full-field condition, spheres were spread across the scene, whereas in the upper and lower field conditions, the spheres were distributed only within the upper or lower area of the viewpoint, respectively (Figure 1). The total number of spheres was constant across field conditions, and therefore, the local density of the cloud was higher in the upper and lower field conditions than the full-field condition. To prevent participants from dodging the colliding spheres, the spheres were not distributed within 2-m radius from the center of the viewpoint. The cameras simulating the participant’s binocular viewpoints moved either forward or backward, at a constant velocity of ±9.4 m/s, presenting expanding or contracting optic flow to each eye with binocular disparities. The spheres were always shown within a clipping distance of 0.01 to 22 m away from the viewpoint. Once a sphere exceeded the clipping distance, it reappeared randomly at the opposite edge of the range. Approximately 520 spheres were always visible within the headset’s FoV. The retinal size of the spheres continually changed from 0.5° to 2.4° as they approached or receded from the viewpoint.

### Procedure

Before the main experiment, we corrected the vertical FoV of each participant that could be asymmetric in size. Participants sat on a stool and fixated the vertical arrow (2.8°) at the center of the display, which indicated the direction of an upcoming target. The bottom edge of the target, a horizontal white bar spreading through the horizontal FoV, was set at 0.2 m horizontally from and 0.3 m vertically above or below the viewpoints. Once the task began, the target moved vertically toward the center of the display at a constant velocity of 0.01 m/s. The participant pressed a button of the gamepad as the target became visible. The moving direction of the target was alternated in each trial. The task was repeated 15 times for each coming direction. We calculated the mean response distance of the target from the center of the display for each direction. Then, two black blind bars were placed on both the upper and lower edge of the displays, limiting the vertical FoV to the shorter responded distance for both upper and lower visual fields. Once the FoV was calibrated, the participants were asked to stand up, and the stool was removed. Participants stood with their feet together and were instructed to be as still as possible during the stimulus observation. They held the gamepad in both hands in front of their body. When the participants were ready, the main experiment started.

The experiment consisted of three blocks of 12 trials. Each block included two trials for each of the two optic flow directions and three field locations. Each condition was repeated six times in total. The order of stimulus presentations was randomized within each block. Before the main experiment, participants practiced the task required in the main experiment for several trials, until they were familiar with it.

A dark background (identical to the optic flow background) was presented for 5 s before each trial to reduce residual effects from the previous trial. Then, two white disks (with 3.3° and 1.8° diameters each) appeared at the center of the displays. The former was fixed at the center of the VR scene, and the latter was used as a heading pointer fixed at the center of the displays. Participants were instructed to hover the pointer over the former disk by moving their head to orient their viewpoints straight ahead. When the participant succeeded in keeping the angular distance of the centers of the two disks at less than 1° for 3 s, the disks disappeared and the next trial started. After presenting only the dark background for a random duration of 2 to 3 s, the optic flow stimulus was presented for 10 s. Participants were instructed to fixate on the blue fixation cross (1.3°) at the center of the display during the stimulus presentation. During stimulus observation, they were also instructed to evaluate whether they felt vection, and how compelling the vection was, for the rating task afterward. If they moved their head more than 3° from the upfront, a beep warned participants through the headphones. To mask room noise that could provide positional cues, pink noise of approximately 62 dB SPL was presented through the headphones during the optic flow presentation.

After each stimulus presentation, participants were asked to report the overall strength of vection ranging from 0 to 100 using a directional pad. A value of 0 meant that the participant felt completely stationary throughout the presentation. A value of 100 meant that the participant felt very strong vection (Seno, Ito, Sunaga, & Nakamura, 2010).

## Results

Vection and head sway data were analyzed by repeated analyses of variance to identify the main and interaction effects of optic flow direction and flow locations. Familywise errors were corrected with Shaffer’s modified sequentially rejective Bonferroni procedure, and the adjusted *p* values are shown for the post hoc analyses. We used an open source function library working on R software named *Anovakun* for the statistical analysis (http://riseki.php.xdomain.jp/index.php?ANOVA%E5%90%9B).

### Vection Strength Ratings

Despite the relatively short presentation of 10 s, most of the test trials exceeded zero rating, except for two trials by two participants with zero rating.

**Figure 2.**
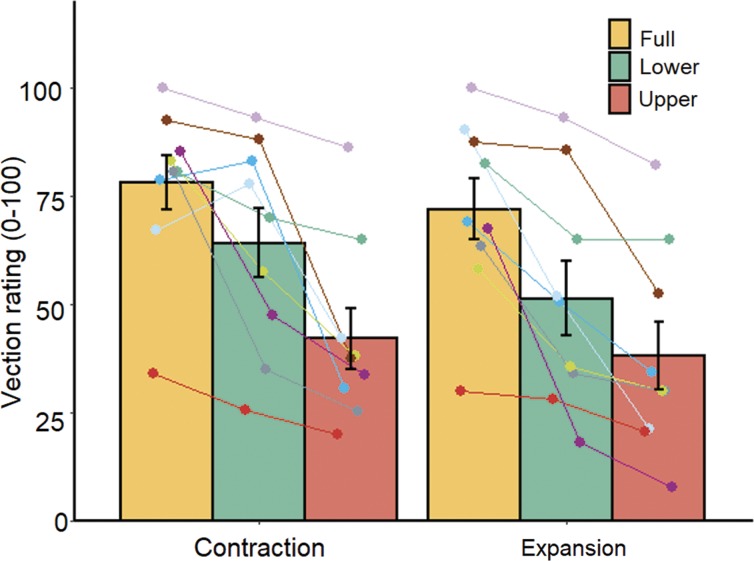
Mean vection strength ratings for each location and direction of optic flow. Error bars represent standard error of the mean. Each line represents data from an individual participant.

Figure 2 shows the mean vection strength ratings across participants under the two optic flow conditions for the three flow location conditions. There were significant main effects of optic flow, *F*(1, 8) = 6.17, *p* = .038, η^2^ = 0.021, and flow location, *F*(2, 16) = 23.85, *p* < .001, η^2^ = 0.303. The two-way interaction of optic flow and flow location was not significant, *F*(2, 16) = 1.23, *p* = .318, η^2^ = 0.005. Multiple comparisons of flow location revealed stronger vection ratings of lower field versus upper field condition, *t*(8) = 3.62, *p* = .007, and stronger vection ratings of full-field versus upper, *t*(8) = 6.53, *p* = .001, and lower field conditions, *t*(8) = 3.50, *p* = .008.

### Head Displacement

**Figure 3.**
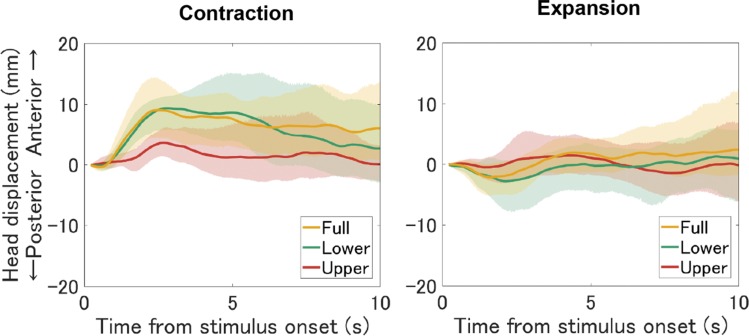
Mean head displacement trajectories relative to the base position across all participants for each flow location and direction. Lines represent average head position across trials in each condition. Ribbons represent the 95% confidence intervals for each data point, obtained by the bootstrap method. Positive and negative values on the vertical axis represent anterior and posterior displacement, respectively.

**Figure 4.**
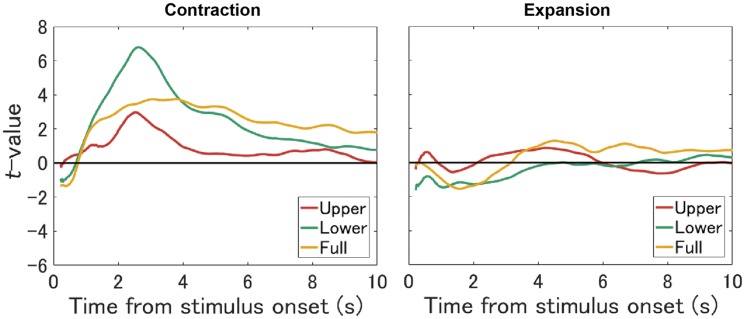
Time series of sample-specific *t* values from the base position across participants for each flow location and direction. Positive and negative values on the vertical axis represent the head moving anteriorly or posteriorly relative to the base position, respectively.

Before the analysis, we discarded the sampled head positions in the first 10 frames (200 ms) from stimulus onset to cope with the frame rate drop at the beginning of each presentation. Figure 3 shows the mean time series of anterior–posterior head sway obtained from the HMD position across participants relative to the initial HMD position of the stimulus presentation (base position). The 95% confidence intervals of each sampling point were computed by a bias-corrected and accelerated (BCa) bootstrap method (Efron, 1987). Contracting stimuli appeared to produce forward head sway, while expanding stimuli appeared to produce slight backward sway. Moreover, the amplitude of the head sway appeared different across flow locations at least for the contracting pattern. Specifically, for the contracting flow, lower and full-field conditions tended to produce larger forward head sway compared with the upper field condition. It is also notable that mean head displacement reached asymptote in the early period of stimulus presentation for both expansion and contraction, with increasing confidence intervals over time. To visualize the time course of the head displacement more clearly, we plotted *t* values as an indicator of deviation from the base position as a function of time for each condition (Figure 4). The *t* values are more remarkable in the early period of stimulus presentation, gradually returning to baseline in the late period, possibly because of sway back due to nonvisual (e.g., vestibular) signals. We therefore divided the head sway data into the first 5 s (early period) and the last 5 s (late period) and analyzed them separately. Note that this decision to split the data was post hoc.

We calculated the head displacement bias for each trial as the mean head displacement during optic flow presentation relative to the base position. Head displacement bias (“head displacement”) for each trial was defined as follows:

(1) $$\text{head}\;\text{displacement}=\frac{1}{N}\overset{N}{\underset{n}{\sum }}x_{n}\;$$

where *x* is the head position for each sampled point, and *N* is the number of sampled data points.

We also calculated the absolute value of head displacement to analyze the magnitude of the head displacement. The absolute head displacement for each trial was defined as follows:

(2) Absolute head displacement=|head displacement|

We then averaged head displacement and absolute head displacement for each participant over six trials per condition.

**Figure 5.**
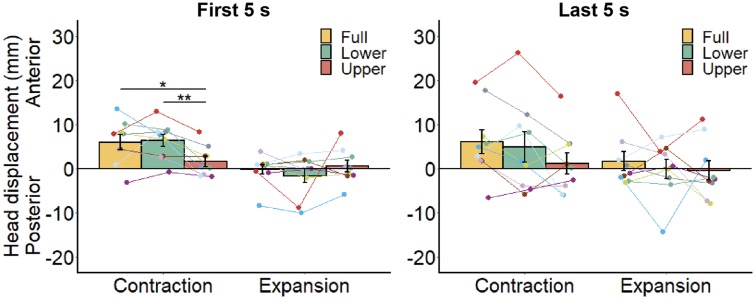
Mean head displacement relative to the base position for each location and direction of optic flow. We separately calculated the mean head displacement for (a) first 5 s and (b) last 5 s from the stimulus onset. Each line represents data from an individual participant. Error bars represent standard error of the mean. *p < .05, **p < .01.

The bar plots in Figure 5 show the mean head displacement across all participants during early and late periods of the stimulus presentation under the two optic flow conditions and the three flow location conditions. In the early period, there was a significant main effect of optic flow direction, *F*(1, 8) = 8.14, *p* = .021, η^2^ = 0.262, indicating different biases in head displacement for contraction and expansion, but no significant main effect of flow location, *F*(2, 16) = 1.78, *p* = .200, η^2^ = 0.024. There was a significant interaction of optic flow and flow location, *F*(2, 16) = 6.07, *p* = .011, η^2^ = 0.089. Subsequent analysis revealed significant simple main effects of optic flow for full-field, *F*(1, 8) = 6.78, *p* = .031, η^2^ = 0.374, and lower field, *F*(1, 8) = 10.21, *p* = .013, η^2^ = 0.476, conditions but not for the upper field condition, *F*(1, 8) = 0.57, *p* = .474, η^2^ = 0.021. There was a simple main effect of flow location for the contracting pattern, *F*(2, 16) = 7.45, *p* = .005, η^2^ = 0.225, but not for the expanding pattern, *F*(2, 16) = 1.14, *p* = .345, η^2^ = 0.054. Multiple comparisons of the flow location for contraction revealed larger head displacement in lower and full-field conditions than the upper field condition, *t*(8) = 4.20, *p* = .009; *t*(8) = 2.75, *p* = .025, but no significant difference between lower field and full-field conditions, *t*(8) = 0.26, *p* = .803. In the late period, there was a significant main effect of flow location, *F*(2, 16) = 4.25, *p* = .033, η^2^ = 0.036, whereas no significant main effect of optic flow, *F*(1, 8) = 2.82, *p* = .132, η^2^ = 0.059, or two-way interaction, *F*(2, 16) = 0.473, *p* = .632, η^2^ = 0.010, was observed. Multiple comparisons revealed larger head displacement in the full-field versus upper field condition, *t*(8) = 3.02, *p* = .049, but no significant difference between upper and lower field conditions, *t*(8) = 1.88, *p* = .097, or full and lower field conditions, *t*(8) = 1.08, *p* = .313.

**Figure 6.**
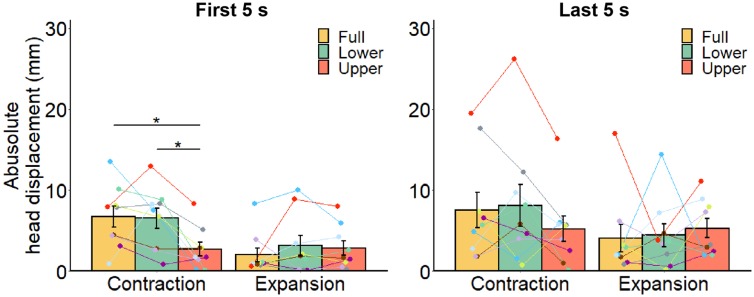
Average absolute head displacements relative to the base position for each location and direction of optic flow. As in Figure 3, we separately calculated the mean absolute head displacement of (a) first 5 s and (b) last 5 s from the stimulus onset. Each line represents data from an individual participant. Error bars represent standard error of the mean. *p < .05.

The bar plots in Figure 6 show the mean absolute head displacement across all participants during early and late periods of the stimulus presentation under the two optic flow conditions and the three flow location conditions. In the early period, there was a significant main effect of optic flow, *F*(1, 8) = 10.58, *p* = .012, η^2^ = 0.134, but not the flow field, *F*(2, 16) = 2.37, *p* = .125, η^2^ = 0.060. There was a significant interaction of optic flow and flow location, *F*(2, 16) = 8.27, *p* = .003, η^2^ = 0.080. There were significant simple main effects of optic flow for full-field, *F*(1, 8) = 18.46, *p* = .003, η^2^ = 0.364, and lower field, *F*(1, 8) = 9.12, *p* = .017, η^2^ = 0.185, conditions but not for the upper field condition, *F*(1, 8) = 0.02, *p* = .891, η^2^ = 0.001. There was also a simple main effect of flow location for the contracting pattern, *F*(2, 16) = 5.52, *p* = .015, η^2^ = 0.241, but not the expanding pattern, *F*(2, 16) = 0.76, *p* = .482, η^2^ = 0.026. Multiple comparisons of flow location for the contraction revealed larger absolute head displacements in lower and full-field conditions versus the upper field condition, *t*(8) = 3.44, *p* = .026; *t*(8) = 2.52, *p* = .036, but no significant difference between the lower field and full-field conditions, *t*(8) = 0.13, *p* = .904. In the late period, there were no significant main effects—optic flow: *F*(1, 8) = 2.35, *p* = .164, η^2^ = 0.048; flow location: *F*(2, 16) = 0.40, *p* = .679, η^2^ = 0.006, or interaction, *F*(2, 16) = 1.23, *p* = .319, η^2^ = 0.026, although the overall pattern of results did not differ drastically from the early period.

## Discussion

### Summary of Results

We examined whether optic flow presented in the lower visual field has a predominant role in postural control, as previously suggested (Agathos et al., 2017; Baumberger et al., 2004; Flückiger & Baumberger, 1988; Gibson, 1950; Previc, 1998; Tamada & Seno, 2015) but not directly confirmed. Here, we demonstrate that optic flow presented in the lower and full visual fields produced larger head displacement than in the upper visual field, although statistical significance was limited to the contracting optic flow. We also found that, in the early period of stimulus presentation, the influence of optic flow direction on head displacement was statistically significant for full and lower field conditions but not for the upper field condition. These findings support the hypothesis that the lower visual field has a stronger influence on postural control than the upper visual field. In addition to the main finding, we found stronger vection with the presentation of lower versus upper field optic flow, consistent with previous studies (Sato et al., 2007; Telford & Frost, 1993). Full-field optic flow induced stronger vection than upper and lower field optic flows.

Note that some nonsignificant results could be due to the relatively small sample size. For example, for head movement, the effect of flow direction in the upper visual field or the effect of visual field with expanding flow might emerge more clearly with larger samples. The effect sizes, however, appear to be small, and any such effects are not crucial for our main findings.

### Ecological Relevance

An ecological perspective can explain the predominant role of the lower visual field on postural control (Gibson, 1950; Previc, 1998), as objects near the ground and hence the lower visual field may give more reliable information about self-motion and orientation than objects above eye-level, in the upper visual field (Gibson, 1950). Although some authors have referred to such an ecological account (Agathos et al., 2017; Baumberger et al., 2004; Flückiger & Baumberger, 1988; Gibson, 1950; Previc, 1998; Tamada & Seno, 2015), no previous study has directly examined the effect of visual location of the optic flow. As noted in the Introduction section, Flückiger and Baumberger (1988) argued that optic flow projected at ground level produces more rapid body sway than optic flow projected at other visual locations, based on the comparison with another study (Lestienne et al., 1977) that had a different experimental setup. The current study has shown superiority of the lower visual field over the upper visual field for postural sway, using identical equipment and stimuli between conditions. Our findings provide experimental evidence that optic flow near the ground provides more reliable information for postural control. Note, however, that it is still unknown whether the upper visual field simply has a weak role in inducing head displacement and vection, or an inhibitory role. No evidence for inhibition was found in the present study, as the full field induced stronger vection than the lower visual field alone.

Larger head displacement under lower field versus upper field stimulation could be related to the proximity of the optic flow from the body under daily conditions. Because our stimuli had a tunnel-like structure, we simulated the viewpoint aligned to the center of the stimulation area, making sure that the distance between the viewpoint and the optic flow was equal in both fields. However, the optic flow elements were actually closer to the lower limbs in the lower field condition than in the upper field condition because the stimuli were centered to the eyes, hence near the top of the body. Although the influence of the vertical proximity on VEPR is unknown, some studies have shown that horizontal proximity to the observer enhances VEPR (Barela, Sanches, Lopes, Razuk, & Moraes, 2011; Godoi & Barela, 2008; Kapoula & Lê, 2006; Lê & Kapoula, 2006; Moraes, De Freitas, Razuk, & Barela, 2016; Moraes, Lopes, & Barela, 2009).

### Attentional Modulation

One might argue that our results could be mediated by attentional bias toward the lower visual field (Dobkins & Rezec, 2004; He et al., 1996), although it has been controversial whether lower visual field has attentional or perceptual/sensory bias (Carrasco et al., 1998, 2006; Talgar & Carrasco, 2002). As both VEPR (Kuno et al., 1999) and vection (Seno, Ito, & Sunaga, 2011) require attention to the visual motion, the present results could have been affected by more attention to the optic flow in the lower than the upper visual field. However, it is also reported that vection is predominantly induced by optic flow that goes unattended (Kitazaki & Sato, 2003), suggesting that non-attended-to stimulation is more important for self-motion perception. As attentional modulation has various aspects, it is still an open question how VEPR and vection are enhanced by attention to the lower visual field.

### On Expanding Flow

We did not find a significant effect of flow location on head displacement for the expanding pattern. These results are similar to Holten, Donker, Verstraten, and Van Der Smagt (2013), who revealed the effect of visual speed and rigidity on CoP sway for contracting optic flow but also expanding optic flow. Wei, Stevenson, and Kording (2010) also found limited effects of optic flow speed on the CoP sway for expansion. This could be simply because the participants were less sensitive to expanding optic flow than to contracting optic flow (M. Edwards & Badcock, 1993; M. Edwards & Ibbotson, 2007; Raymond, 1994), although there are some contradictory results (Ball & Sekuler, 1980; Shirai & Yamaguchi, 2004). Another explanation could be that the biomechanical constraint was more severe for pitching backward than forward, as our feet are attached forward and easy to control swaying forward, when compared with backward (M. Edwards & Ibbotson, 2007; Holten et al., 2013; Lestienne et al., 1977; Palmisano, Pinniger, Ash, & Steele, 2009). Backward sway was possibly inhibited by the biomechanical constraint, also obscuring the postural effects of flow location. It remains to be examined, however, whether the expansion simply produced similar neural responses for postural control across flow location conditions or the postural responses were inhibited by other compensating mechanisms including biomechanical constraint. The latter explanation could be more plausible because vection was sufficiently induced even in the expansion, and there was clear influence of flow location on vection strength in the expansion, which indicates different sensory signals across the flow locations.

### Sway Dispersion in the Late Period

Head sway varied across participants in the late period as shown in Figures 4 and 5, probably reflecting individual differences in sway amplitude and frequency, which may in turn contribute to individual differences in the dominance of nonvisual inputs indicating body inclination caused by optic flow. Studies have shown that maintaining upright posture is achieved by dynamically integrating visual, vestibular, and proprioceptive inputs based on their reliability (Goodworth, Mellodge, & Peterka, 2014; Nashner & Berthoz, 1978; Peterka, 2002; Peterka & Benolken, 1995; Peterka & Loughlin, 2004), named sensory reweighting (Nashner & Berthoz, 1978). Greater variation in postural sway in the late period could be due to individual differences in the sensory reweighting parameters, including feedback delay, or changing sensory weights among modalities.

### Vection and VEPR

Research has shown that vection and VEPR are closely related, implying shared neural mechanisms. For example, the magnitude of postural sway increases during self-motion perception compared with object-motion perception (Freitas Júnior & Barela, 2004; Tanahashi et al., 2007; Thurrell & Bronstein, 2002). It is suggested that there are short- and long-latency mechanisms underlying VEPR, the former evoking automatic postural adjustment and the latter enhancing VEPR during self-motion perception (Guerraz & Bronstein, 2008). Several recent studies have shown that spontaneous postural sway can predict individual vection strength (Apthorp, Nagle, & Palmisano, 2014; Palmisano, Apthorp, Seno, & Stapley, 2014; Palmisano, Arcioni, & Stapley, 2018).

With respect to the current study, vection strength and the magnitude of VEPR change consistently with changes in visual intensity such as the occluding area (Kawakita et al., 2000), stimulus speed (Kuno et al., 1999; Lestienne et al., 1977), and stimulus density (Lubeck et al., 2015).

In the present study, effects of flow location on vection strength were partly consistent with effects on head displacement. Lower field optic flow induced stronger vection than upper field optic flow, consistent with previous work (Sato et al., 2007; Telford & Frost, 1993). Similarly, we found that lower field optic flow produced larger VEPR than upper field optic flow, although the effects of flow location were limited to the early period of contraction. Our findings for upper and lower field conditions are in line with previous studies, indicating consistent response to visual stimuli in vection and postural sway (Kawakita et al., 2000; Kuno et al., 1999; Lestienne et al., 1977; Lubeck et al., 2015).

Results regarding vection and head displacement in full-field and lower field conditions may appear inconsistent. Full-field optic flow induced stronger vection than lower field optic flow, which indicates the effect of stimulus size or stimulus occlusion on vection (Brandt et al., 1973; Nakamura, 2001). As for head displacement, we did not find a significant difference between the lower and full-field conditions. This could be due to a ceiling effect reflecting limited body inclination, while vection would not have suffered from that limitation. This partial inconsistency between vection and VEPR in the effects of flow location implies that postural control and vection are differently modulated in part, in accordance with previous studies (Previc, 1992; Previc & Mullen, 1991), even though they should share some mechanisms as indicated by the different head displacements across upper and lower conditions.

Contracting optic flow induced stronger vection than expanding optic flow, also producing larger head displacement. These results support the previous studies that found stronger vection for a contracting pattern than an expanding pattern (Berthoz et al., 1975; Bubka, Bonato, & Palmisano, 2008; Ito & Shibata, 2005; Seno et al., 2010) in both children and adults (Shirai, Endo, Tanahashi, Seno, & Imura, 2018), as well as postural sway (Holten et al., 2013; Lestienne et al., 1977; Palmisano et al., 2009). A well-known hypothesis for the superiority of the contracting pattern is the directional anisotropy of the locomotion experience (Bubka et al., 2008). The sensory integrating system accumulates the exposure history of past sensory inputs and compares incoming inputs to the previous ones (Held, 1961). Bubka et al. (2008) argued that our greater exposure to moving forward makes the vestibular system expect forward acceleration on the presentation of the expanding pattern, and self-motion perception is inhibited without vestibular inputs. By contrast, the contracting pattern without vestibular inputs is less inconsistent with our exposure history and therefore could induce stronger vection than expanding optic flow. The same mechanism also explains larger VEPR for the contracting pattern than for the expanding pattern (Holten et al., 2013; Palmisano et al., 2009).

Optokinetic nystagmus (OKN) mechanisms might be also involved in the contraction superiority in vection. Vection and OKN might share neural mechanisms, especially in the vestibular nuclei (Brandt, Dichgans, & Büchele, 1974), as their characteristics correspond well with each other (Brandt et al., 1973, 1974; Flanagan, May, & Dobie, 2002; Schor, Lakshminarayanan, & Narayan, 1984; Seya, Shinoda, & Nakaura, 2015). Seno et al. (2010) and Seno and Sato (2009) found that temporonasal motion on the nasal retina, which is included in the contracting optic flow, induces stronger vection than the nasotemporal motion or motion in the temporal retina. Based on the previous report that the same type of retinal motion also produces strong OKN (Murasugi, Howard, & Ohmi, 1986), Seno et al. (2010) suggested the involvement of OKN mechanisms in the contraction superiority on vection. To our knowledge, the relationship between OKN and VEPR has not been directly examined, but our finding of larger head displacement for the contraction than expansion might be attributed to OKN mechanisms as well as vection.

## Conclusion

Here, we demonstrated that optic flow presented in the lower visual field produced larger head displacement than optic flow presented in the upper visual field. Vection was stronger under the presentation of the lower field optic flow compared with the upper field optic flow, in accordance with head displacement. Our findings suggest that ecologically relevant visual cues have a predominant role in postural control, as well as in the perception of self-motion.
